# Transient Receptor Potential Ankyrin 1 (TRPA1) Methylation and Chronic Pain: A Systematic Review

**DOI:** 10.3390/genes14020411

**Published:** 2023-02-04

**Authors:** Fulvio Celsi, Francesca Peri, Julia Cavasin, Luisa Zupin, Giorgio Cozzi, Egidio Barbi, Sergio Crovella

**Affiliations:** 1 Institute for Maternal and Child Health-IRCCS Burlo Garofolo, 34137 Trieste, Italy; 2 Department of Medicine, Surgery, and Health Sciences, University of Trieste, 34127 Trieste, Italy; 3 Biological Science Program, Department of Biological and Environmental Sciences, College of Arts and Sciences, Qatar University, Doha 2713, Qatar

**Keywords:** pain, methylation, TRPA1

## Abstract

Background and Objective: Chronic pain represents a major global health issue in terms of psycho-physiological, therapeutic, and economic burden, not limited to adults but also to the pediatric age. Despite its great impact, its molecular mechanisms have still not been completely unraveled. Focusing on the impact of epigenetics in the pain complex trait, we assessed the association between chronic pain and the methylation pattern of TRPA1, a key gene related to pain sensitivity. Methods: We conducted a systematic review retrieving articles from three different databases. After deduplication, 431 items were subjected to manual screening, and then 61 articles were selected and screened again. Of these, only six were maintained for meta-analysis and analyzed using specific R packages. Results: Six articles were divided into two groups (group 1: comparison of mean methylation levels between healthy subjects and patients with chronic pain; group 2: correlation between mean methylation levels and pain sensation). A non-significant mean difference was obtained from the analysis of group 1 with a value of 3.97 (95% C.I. −7.79; 15.73). Analysis of group 2 showed a high level of variability between studies (correlation = 0.35, 95% C.I. −0.12; 0.82) due to their heterogeneity (I^2^ = 97%, *p* < 0.01). Conclusions: Despite the high variability observed in the different studies analyzed, our results suggest that hypermethylation and increased pain sensitivity could be connected, possibly due to the variation of TRPA1 expression.

## 1. Introduction

Chronic pain is a growing health issue and a major psychological and socioeconomic burden affecting a significant proportion of the population worldwide [1]. As a matter of fact, the prevalence of chronic pain in 2019 was 20.4%, with the highest impact among women and those aged 65 and over (https://www.cdc.gov/nchs/products/databriefs/db390.htm) (accessed on 15 November 2022). Children can be affected too. Indeed, it has been estimated that 20–35% of children worldwide experienced chronic pain.

Patients with cancer or other chronic disease are more prone to experience chronic pain, for example, in a post-operative context or after a long-term post-traumatic recovery. Recurrent pains, non-associated with a specific disease, are very common; indeed, a prevalence rate of 8–83% for headache, 4–53% for abdominal, 4–40% for musculoskeletal, and 14–24% for back pains were reported by King et al. [2]. Despite this high prevalence, this topic in the pediatric field has not been systematically reviewed after the comprehensive work by King et al., highlighting the need for an update [3]. To note, the range of prevalence varied between the studies, indicating that the real impact of this health issue is uncertain but probably underestimated. Chronic pain can be persistent, episodic, or cyclical with a duration greater than 3 months; nevertheless, given its long-lasting nature, it clearly requires regular and repeated clinical evaluations and measures with quantitative and qualitative tools [4]. The evaluation may be complicated by the difficulty in the clinical assessment of pain in the youth or in patients with cognitive impairment [2]. 

A detailed evaluation helps define a tailored treatment whose efficacy is often limited and requires a non-pharmacological approach [5]. This is secondary to the complex nature of chronic pain triggers and enduring mechanisms, a field of research still not fully exploited [6]. 

Pain sensation begins with the activation of a variety of receptors in response to mechanical, chemical, or thermal stimuli, followed by the generation of action potentials in the peripheral nociceptors and their transmission through various types of sensory neurons from the periphery towards the central nervous system, each with its own signaling pathway. Keeping in mind that pain is a complex and multifactorial trait, several molecules involved in nociception pathways have been investigated. The transient receptor potential (TRP) channels, a group of ion channels expressed in sensory neurons and activated by different chemical substances and irritants, are among the most studied. Different members of the group were previously described, as the TRP Vanilloid (V) 1, a thermosensitive and nociceptive receptor, also plays a role in inflammatory hyperalgesia. TRPV1 can be modulated by bradykinin via prostaglandin receptor E2 (EP4) [7]. Moreover, a cross-talk between TRPV-1 and adenosine receptors (AR), probably through G-protein and c-AMP signaling pathway, was also described, and some adenosine ligands present analgesic activity modulating TRPV1 activity [8]. Intriguingly, the G-protein-cAMP pathway was also a mediator between cannabinoid receptors and TRPV1, showing a pleiotropic role of this signaling in neurons [9]. 

Another member of the TRP channels is the transient receptor potential ankyrin 1 (TRPA1), which can be mechano- or thermosensitive. TRPA1 is categorized as a non-selective cation channel, with a strong preference towards divalent cations (Ca^2+^/Mg^2+^) [10], and it is expressed in several tissues. TRPA1 was originally identified in human fibroblasts [11] and subsequently in nociceptive neurons [12]. It is also widely expressed in the gastrointestinal system, where it can regulate enteric function through serotonin release from enterochromaffin cells [13]. Animal studies show that TRPA1 expression can be induced by oxidative stress in spinal cord dorsal horns [14], probably mediating central hyperalgesia. Furthermore, in a model of neuropathic pain (induced by nerve ligation), TRPA1 expression is found to be reduced in ligated nerves, but it is increased in intact surrounding fibers [15]. Inflammatory stimuli can increase TRPA1 expression in lung cells, in particular Tumor Necrosis Factor-α (TNF-α) acting synergistically with interleukin-1β (IL-1β) and interferon-γ (IFN-γ) [16]. These studies suggest that a variety of stimuli can be associated with the induction of the TRPA1 gene, resulting in an increase in nociception. 

Recent studies showed that chronic pain can be related to changes in DNA methylation, the prototypical epigenetic mechanism, leading to the modulation of pain pathways from the spinal cord to the cortex [17,18]. Moreover, methylation studies have explored epigenetic modulation in several chronic pain conditions [19,20,21], proving that these alterations can affect pain perception and evoke pathological pain responses [22]. Notably, DNA methylation of TRPA1 in blood cells was shown to be associated with pressure or heat pain in healthy humans [22,23,24] but also with neuropathic pain in chronic pain patients [25]. Mostly in animal studies, it has been proven how these changes can be reversed or modified by targeted therapy offering new promising therapeutical perspectives [26,27].

The aim of this meta-analysis was to investigate the association between TRPA1 methylation and chronic pain conditions in human subjects in adult and pediatric studies.

## 2. Materials and Methods

### 2.1. Search Strategy

**Figure 1:**
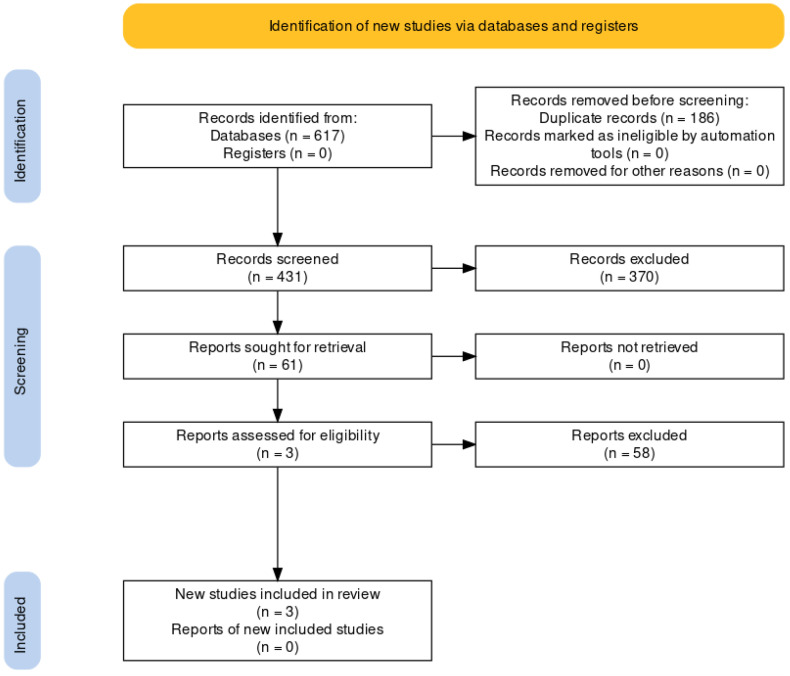
PRISMA flow diagram of the procedures made to extract the works evaluated.

“Litesearchr” R package [28] was used to automatize and broad search terms. First, a “naïve” search was performed on Pubmed, by using the terms “TRPA1” and “methylation” and “pain”, from 2006 (first published report) resulting in 62 journal articles (as of July 2021). In 28 of these items, keywords were indicated by the authors and were then extracted, while for the remaining 34 articles, keywords were inferred from titles, using a method that approximates rapid automatic keyword extraction algorithm (RAKE) (in Litesearchr package). Both lists were merged, obtaining 37 different terms. Subsequently, a matrix was created by counting the recurrence of computed keywords in abstract and titles of each article from the original search. This matrix was then used to perform a network analysis, assessing the “strength” of linkage between each different term. Keywords were then selected having the higher “strength” of association, and these keywords were used to determine a common search string. This string was used in three different databases, PubMed, Web of Science, and Cochrane Library, retrieving 324, 280, and 13 items, respectively (Figure 1). This strategy was performed to avoid bias in keyword selection and article retrieval.

### 2.2. Document Analysis

Deduplication was then performed, removing 186 articles; 431 items were subjected to manual screening. In parallel, a topic analysis was performed (using “revtool” packet for R [29]) to determine whether articles cluster around defined topic. As can be seen in Supplementary Figure S1, articles do not cluster in specific topics/arguments, and manual screening of titles was performed. Studies performed either on animals or in in vitro cellular models as well as pharmacological studies were excluded (primary exclusion criteria); the screening was performed by F.P., J.P., and F.C. independently; results of this primary screening were discussed between those authors to avoid selection bias. After this primary assessment, 61 articles were retrieved and screened. The inclusion criteria were clinical studies with human subjects for article; based on these criteria, only six studies were maintained for the meta-analysis (Figure 1).

### 2.3. Data Extraction and Analysis

Mean methylation level on TRPA1 promoter was the main variable taken into consideration. An RStudio package, called “metaDigitise” [30] was employed to extract raw numerical data from articles when authors have not disclosed them. Data from each study were extracted and subjected to analysis using “Esc” package for effect size calculation and “meta” and “dmetar” for metanalysis [31]. Mean and standard error from each study group were pooled and used to calculate effect size (Hedges’ g) and standard error. Weights were then assessed, as the inverse of standard error and used to determine pooled effect size. Standardized mean difference (SMD) was assessed using “metafor” r package and reported. “Metacont” function from “meta” package was used to generate a random-effect model for data analysis, considering that population subjected to test is considerably heterogeneous. Heterogeneity of studies subjected to metanalysis was assessed using restricted maximum likelihood (REML) procedure. Influence of each study on heterogeneity was examined using “InfluenceAnalysis” function from “dmetar” package and visualized using the “plot” function of the same package.

### 2.4. Risk of Bias Assesment

**Figure 2:**
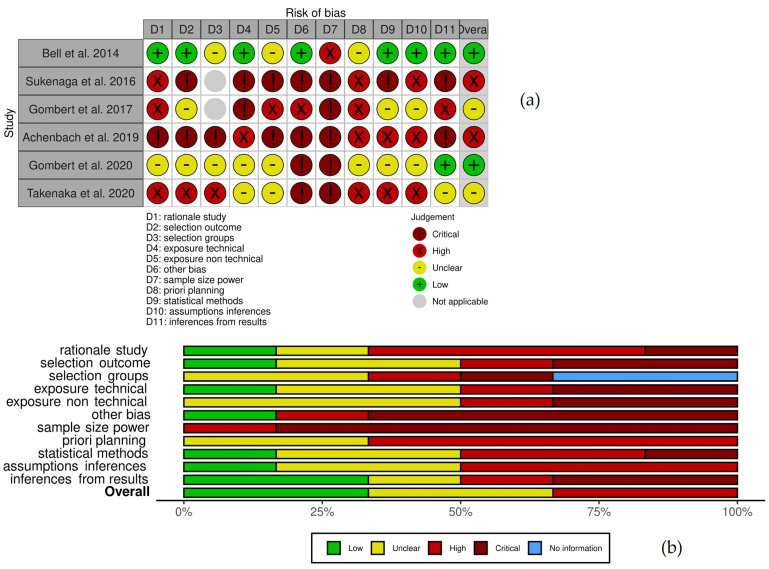
Evaluation of the risk of bias using the Q-Genie tool and presented as single and aggregated results. (**a**) risk of bias in each separated study presented for each single item, and overall (last column, Overall). (**b**) Aggregated results for every single item describing the risk of bias for the studies examined.

As the relevant studies are mainly similar to genetic association studies, to assess risk of bias we employed a specific tool developed to evaluate the quality of genetic studies [32]. This tool aims to instate an instrument for a convenient evaluation of risk of bias in genetic association studies and consist of 11 items assessing different aspects of genetic studies, on a scale from 1 (poor) to 7 (excellent). Those items consider the following various aspect of genetic studies: the scientific basis for development of the research question, ascertainment of comparison group (cases versus control), classification of the tested genetic variant, classification of the outcome, discussion of source of bias, appropriateness of sample size, description of planned statistical analysis, used statistical methods and appropriate interpretation of results. This instrument is validated for internal coherence and reliability. Aggregated evaluation results of every single study are presented in Figure 2.

## 3. Results

### 3.1. Identification and Screening

A semi-automatic strategy was employed to expand the “keywords” used for paper research, as described in Materials and Methods. In summary, a total of 617 items were retrieved, and after removing duplicates, 431 were subjected to further analysis. Following that, we manually screened the records obtained, and based on titles, we selected 61 papers to assess eligibility. After a second round of screening based on the abstract, we obtained six articles that were included in the review. These articles were clinical studies in which pain sensations, either in healthy subjects or patients with chronic pain conditions, are correlated with methylation of CpG island in the TRPA1 gene (Figure 1).

Table 1 displays the essential characteristics of examined studies.

### 3.2. Risk of Bias Assessment

Figure 2a displays the cumulative results for assessing the risk of bias in the six papers selected, and it indicates that a low risk of bias is overall present only in two out of the six papers (Bell et al., 2014 and Gombert et al., 2020), while two possess a high risk of bias (Sukenaga et al., 2016 and Achenbach et al., 2019). The majority of bias derives from Item D7: sample size power, and indeed all the studies considered have a low number of subjects examined; Item D8: a priori planning also seems to be the second responsible for the high bias in the studies examined (Figure 2b); for a complete description of the single items, see [32].

### 3.3. Data Analysis

The six articles included in the review were divided into two groups based on the reported data. The first group deals with the comparison of mean methylation levels between healthy subjects and patients with chronic pain [21,23,33], while the second one deals with the correlation between mean methylation levels and pain sensation, either in healthy subjects [22] or both in healthy subjects and patients [24,25]. Meta-analysis was then performed separately between these two groups due to the high heterogeneity of the reported data; the group of articles presenting “confronting” data was named group 1, and the other is group 2.

**Figure 3:**
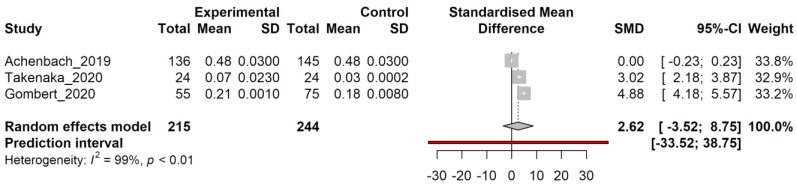
Random effect model analysis of group 1 papers. Study: the first author followed by the year of the study examined. Experimental/Control: data for affected/control groups. Total: number of subjects in each group. Mean: mean value of methylation level for each group. SD: standard deviation. Standardized mean difference: a graph showing the standardized mean differences for each study, with confidence intervals (grey squares). SMD: standardized mean difference value. A 95%-CI: confidence interval for 95% on SMD. Weight: statistical “weight” for each study. In bold characters, results from random effect model analysis are indicated as a graphical diamond on the standardized mean difference graph. Heterogeneity: results from heterogeneity test, I^2^, describes the percentage of variation across studies that is due to heterogeneity rather than chance, *p*: *p*-value derived from Cochran’s Q test.

Analysis of group 1 data started by pooling effect size by standardizing mean difference (SMD) and assessing Hedge’s g effect size for the pooled effect size; restricted maximum likelihood (REML) procedure was employed to determine between-study heterogeneity, corrected by the Knapp–Hartung adjustment. It is noticeable that the high level of heterogeneity (I^2^ = 99%, *p* < 0.01) results in a non-significant pooled SMD with wide confidence intervals (Figure 3).

**Figure 4:**
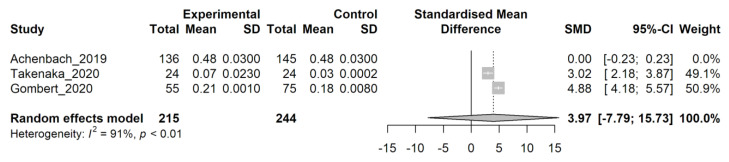
Random effect model analysis of group 1 papers, excluding Achenbach et al., 2019. Study: the first author followed by the year of the study examined. Experimental/Control: data for affected/control groups. Total: number of subjects in each group. Mean: mean value of methylation level for each group. SD: standard deviation. Standardized mean difference: a graph showing the standardized mean differences for each study with confidence intervals (grey squares). SMD: standardized mean difference value. A 95%-CI: confidence interval for 95% on SMD. Weight: statistical “weight” for each study. In bold characters, results from random effect model analysis are indicated as a graphical diamond on the standardized mean difference graph. Heterogeneity: results from heterogeneity test, I^2^, describes the percentage of variation across studies that is due to heterogeneity rather than chance, *p*: *p*-value derived from Cochran’s Q test.

Assessing which study provides the highest variability, an “influence” analysis was performed (Supplementary Figure S2), and from this inspection, the Achenbach study [23] appears to be the one with the highest heterogeneity. The meta-analysis was repeated, excluding this study, and the results are presented in Figure 4. In this case, heterogeneity is reduced (I^2^ = 91%, *p* < 0.01) but still significant, and the standardized mean difference (SMD) has a value of 3.97 (95% C.I. −7.79; 15.73). Although not significant, due to the high heterogeneity, these results (based on the SMD value) may suggest that patients with chronic pain could have increased levels of methylation on the TRPA1 gene; however, these data are not conclusive, and further trials are necessary.

**Figure 5:**
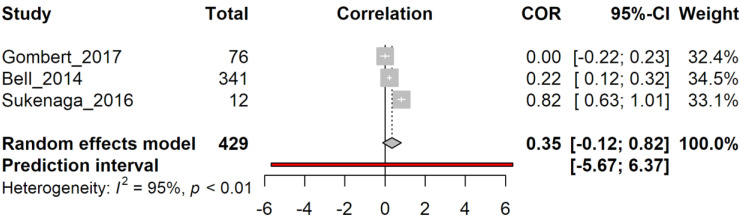
Random effect model analysis of group 2’s papers. Study: the first author followed by the year of the study examined. Total: total number of subjects in each group. COR: correlation (extracted from study) between pain sensations and methylation levels, with confidence intervals (grey squares). A 95%-CI: confidence interval for 95% on COR. Weight: statistical “weight” for each study. In bold characters, results from random effect model analysis are indicated as a graphical diamond on correlation graph. Heterogeneity: results from heterogeneity test, I^2^, describes the percentage of variation across studies that is due to heterogeneity rather than chance, *p*: *p*-value derived from Cochran’s Q test.

Analysis of correlation was then performed on group 2, aiming to determine if a relationship exists between methylation levels on the TRPA1 gene and painful sensation. Figure 5 shows the results of a meta-analysis of the three articles, highlighting a high level of variability within the results (correlation = 0.35, 95% C.I. −0.12; 0.82) due to high heterogeneity (I^2^ = 95%, *p* < 0.01).

**Figure 6:**
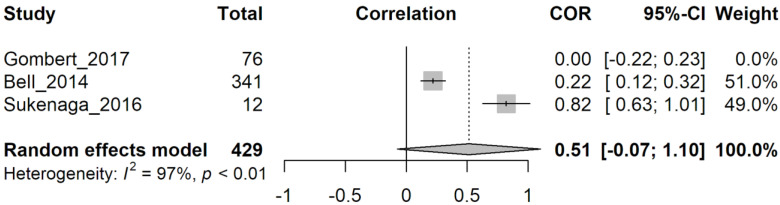
Random effect model analysis of group 2’s papers excluding Gombert (2017). Study: the first author followed by the year of the study examined. Total: total number of subjects in each group. COR: correlation (extracted from the study) between pain sensations and methylation levels with confidence intervals (grey squares). A 95%-CI: confidence interval for 95% on COR. Weight: statistical “weight” for each study. In bold characters, results from random effect model analysis are indicated as a graphical diamond on the correlation graph. Heterogeneity: results from heterogeneity test, I^2^, describes the percentage of variation across studies that is due to heterogeneity rather than chance, *p*: *p*-value derived from Cochran’s Q test.

Moreover, in this case, influence analysis was performed to assess which data show the highest heterogeneity (Supplementary Figure S3). However, no clear-cut results emerged. Then we tried to exclude one work from the analysis because it considered only healthy subjects [22] (Figure 6), but heterogeneity was not reduced (I^2^ = 97%, *p* < 0.01).

**Figure 7:**
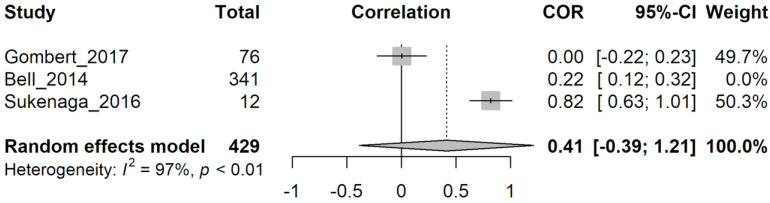
Random effect model analysis of group 2 papers excluding Bell (2014). Study: the first author followed by the year of the study examined. Total: total number of subjects in each group. COR: correlation (extracted from the study) between pain sensations and methylation levels with confidence intervals (grey squares). A 95%-CI: confidence interval for 95% on COR. Weight: Statistical “weight” for each study. In bold characters, results from random effect model analysis are indicated as a graphical diamond on the correlation graph. Heterogeneity: results from heterogeneity test, I^2^, describes the percentage of variation across studies that is due to heterogeneity rather than chance, *p*: *p*-value derived from Cochran’s Q test.

We tried to exclude the less recent study [24] since methylation analysis methods are improved over time. Still, in this case, heterogeneity was not reduced (I^2^ = 97%, *p* < 0.01) (Figure 7). Due to high levels of heterogeneity between the studies in group 2, it is not possible to obtain firm conclusions, but methylation levels appear to correlate slightly with pain sensation.

## 4. Discussion

Bearing in mind the increasing importance and growing awareness of DNA methylation and its mechanisms as a biomarker in health studies, we decided to focus on a key gene related to pain sensitivity, TRPA1.

In this systematic review, we observed that available studies about the role of TRPA1 methylation in pain are scarce, based on different populations and different outcomes, and poorly homogenous. Our final selection resulted in six papers. This result demonstrates the lack of research in linking chronic pain in different settings to TRPA1 methylations’ levels, suggesting that further research is warranted. Furthermore, the experimental setups between these different studies are variable and not standardized. Aiming to assess if it is possible to reduce heterogeneity, we divided the selected articles based on a statistical comparison between methylation levels and pain signatures (different in each research work) as follows: one group based on a comparison between mean (group 1) and the other on a linear regression model of methylation and pain levels (group 2).

Studies from a comparison of mean methylation levels between healthy subjects and patients with chronic pain (group 1) evaluated the relationship between TRPA1 methylation and pain in different settings. Takenaka and coworkers [21] explored this relationship by comparing neuropathic pain in individuals with chronic pain and preoperative patients scheduled for thoracic surgery. The authors found a positive correlation between increased neuropathic pain scores and TRPA1 promoter methylation in patients who suffered chronic pain. Gombert et al. [33] analyzed pain in Crohn’s disease (thus linked to inflammation) in comparison with healthy peers, using also quantitative sensory testing to assess pain sensitivity; Crohn patients presented lower pressure pain threshold in comparison with healthy participants, with inverse correlation in TRPA1 methylation levels. These two studies suggest that chronic pain is probably correlated with increased TRPA1 methylation levels. The third study of group 1 [23] dealt with women with multi-somatoform disorder (MSD), a condition characterized by at least three medically unexplained but bothersome physical symptoms and a history of somatization, with the presence of pain-related symptoms. This research dealt with a peculiar setting, where the pain is secondary to a non-organic disease such as an adverse childhood experience (as stated by the authors), suggesting a more intricate and complex etiology. As a matter of fact, a statistical analysis of heterogeneity denotes this study as a sort of ‘outlier’, and consequently, we decided to exclude it from our meta-analysis. Thus, considering the two retained articles, we observed a putative difference in standardized mean difference with a possible trend toward a higher mean methylation level in patients with neuropathic and inflammatory pain, although without a clear statistical significance.

However, the different triggers of pain in the two studies, one due to an underlying inflammatory disease (Chron disease, [33]) and the other possibly caused by multiple conditions (neuropathy, [21]), may have impacted the results, increasing the variability between them.

Studies investigating the correlation between mean methylation levels and pain sensation (group 2) show similar findings, which, although not always statistically significant, suggest a possible correlation between increased pain sensation and higher levels of the TRPA1 promoter methylation. In the study by Bell and coworkers [24], individual thermal pain sensitivity has been linked to differences both in methylation of TRPA1 gene promoter and gene expression in discordant twins and healthy volunteers. Enhanced methylation was shown to be associated with higher TRPA1 expression in tissues as well as increased thermal pain sensitivity. Examining a small, heterogeneous cohort of patients with chronic pain, Sukenaga et al. [25] observed a positive correlation between enhanced scores on neuropathic pain evaluation and TRPA1 methylation levels. In the latter study by Takenaka and coworkers [21], pressure pain threshold was correlated with TRPA1 methylation levels in healthy participants; the authors found that subjects with hypermethylated TRPA1 display a lower pain threshold. Moreover, in group 2 studies, increased pain sensation can be correlated with hypermethylated TRPA1 gene promoter.

As previously stated, TRPA1 can be activated by a variety of compounds, such as inflammatory mediators, allyl isothiocyanate from mustard oil or wasabi, or diallyl disulfide from garlic [34]. Low temperature can also activate this channel [35], although it directly or through other mechanisms remains under debate [36]. The broad expression of TRPA1 in different tissues could suggest its involvement in different pathways, although several pieces of research identify this channel as important in pain sensation transduction, either in acute or chronic settings. Our metanalysis results gives a similar suggestion, even if the examined studies evaluated a different kind of noxious stimuli in different diseases. Moreover, all these studies dealt only with adult subjects. Studies that have explored the role of methylation in children’s pain sensations are scarce, probably due to the complexity of evaluating nociception in young children. Chidambaran and coworkers [37] suggest that chronic postsurgical pain in children could be linked to specific methylation profiles as follows: a study enrolling 73 adolescents undergoing posterior spine fusion assessed global DNA methylation and found a correlation between chronic post-operatory pain and differential methylation of GABA and the dopamine-DARPP32 pathway, linked to emotion- reward circuit and pain. Early stressful events, such as permanence in neonatal intensive care unit (NICU) as can occur in very preterm infants (VPT), can affect methylation levels in the serotonin transporter gene (SLC6A4), leading to emotional dysregulation and increasing susceptibility for developing behavioral problems in the late age [38,39]. Furthermore, in VPT, the following epigenetic mechanism appears to be in place: hypomethylation of retrotransposons LINE-1 (L1), an active source of genomic mosaicisms, is likely related to neurodevelopmental issues [40]. According to a recent systematic review, the quality of maternal caring can influence the behavioral development trajectories of newborns and children based on data from eleven distinct studies [41]. All these data point out that methylation levels, pain, neurodevelopment, and behavior can be linked in newborns and children.

Keeping in mind the high variability observed in our analysis, the observed trend may suggest that hypermethylation and increased pain sensitivity are connected, possibly due to decreased TRPA1 expression. This may implicate a further role of this channel in nociception as a potential regulator of inflammatory pain [42]. However, our study suffers some limitations. First, there is just a limited number of studies dealing with TRPA1 methylation and pain; second, these studies are quite heterogeneous. Moreover, very little is known about the variability of methylation patterns in children and adolescents with and without chronic pain conditions. There is a gap between the lack of studies in children and the few available data in adults, missing the opportunity of outlining the course of modification in pain reception, transmission, and processing and its impact on cognitive development. Lastly, the distinction between disease-correlated and non-correlated chronic pain conditions has not been adequately investigated. We believe that defining an epigenetic profile in these groups of patients could offer interesting insights into pain origin and treatment.

In conclusion, this review shows that available studies focused on the role of TRPA1 methylation in pain are scarce, based on different populations and different outcomes, poorly homogeneous and comparable, without any study investigating the pediatric population. Considering that 17% of adults affected presented a persistent and continuous history of chronic pain from childhood [43], the study of this issue in children should be mandatory.

Future research within a well-defined study framework of homogeneous populations of individuals with and without a history of chronic pain is necessary. The same purpose should lay the foundations for pediatric studies where greater is the chance to assess the impact of chronic pain conditions, gene methylation patterns, and neurocognitive development.

## Figures and Tables

**Table 1:** Characteristics of studies included in meta-analysis: Authors; Date: Bibliographic references; N° of patients, median age: Number and median age of participants of the study, suffering from a specific condition, as reported from authors, * indicate mean; N° of controls, median age: Number and median age of healthy participants of the study, as reported from authors, * indicate mean; Total subjects: the total number (patients + healthy control) of subjects included in the study; clinical condition: the pathology subject of the study or the test employed to differentiate healthy subjects; methylation analysis technology: the technique employed to assess methylation levels, MeDIP-sequencing = methylated DNA immunoprecipitation sequencing, Illumina 450K = Infinium^®^ HumanMethylation450 BeadChip, direct sequencing = Sanger sequencing; relevance = critical significance of the study, as assessed by heterogeneity analysis and number of participants, * = low, ** = medium, *** = high; group: see text.

Authors	Date	N° of Patients	Median Age	N° of Controls	Median Age2	Total Subjects	Clinical Condition	Methylation Analysis Technology	Relevance	Group
**Bell et al.**	2014	50	62	50	64	100	Heat pain tolerance	MeDIP-sequencing; Illumina 450K	***	2
**Sukenaga et al.**	2016	NA	NA	NA	NA	12	Neuropathic pain	Illumina 450K	*	2
**Gombert et al.**	2017	NA	NA	75	33.6 *	75	Pain threshold determination	Direct Sequencing	**	2
**Achenbach et al.**	2019	151	NA	149	NA	300	multisomatoform disorder with pain	Direct Sequencing	*	1
**Gombert et al.**	2020	55	39.9 *	75	33.5 *	130	Crohn’s Disease	Direct Sequencing	***	1
**Takenaka**	2020	48	NA	NA	NA	48	Chronic Pain	Illumina 450K	**	1

## Data Availability

Scripts used for data analysis and manipulation, as well as originals files of the pictures are stored at Open Science Foundation, as a Registry Item and it can be accessed using the following link https://osf.io/k2fb6 (accessed on 3 Februrary 2023).

## Supplementary Materials

The following supporting information can be downloaded at: https://www.mdpi.com/article/10.3390/genes14020411/s1, Figure S1: Topic analysis; Figures S2 and S3: Influence analysis.
