# Supplementary figures and images for: Transient Receptor Potential Ankyrin 1 (TRPA1) Methylation and Chronic Pain: A Systematic Review
Source: Genes (Basel). 2023 Feb 4;14(2):411. doi: 10.3390/genes14020411 (PMC9957263; doi:10.3390/genes14020411)

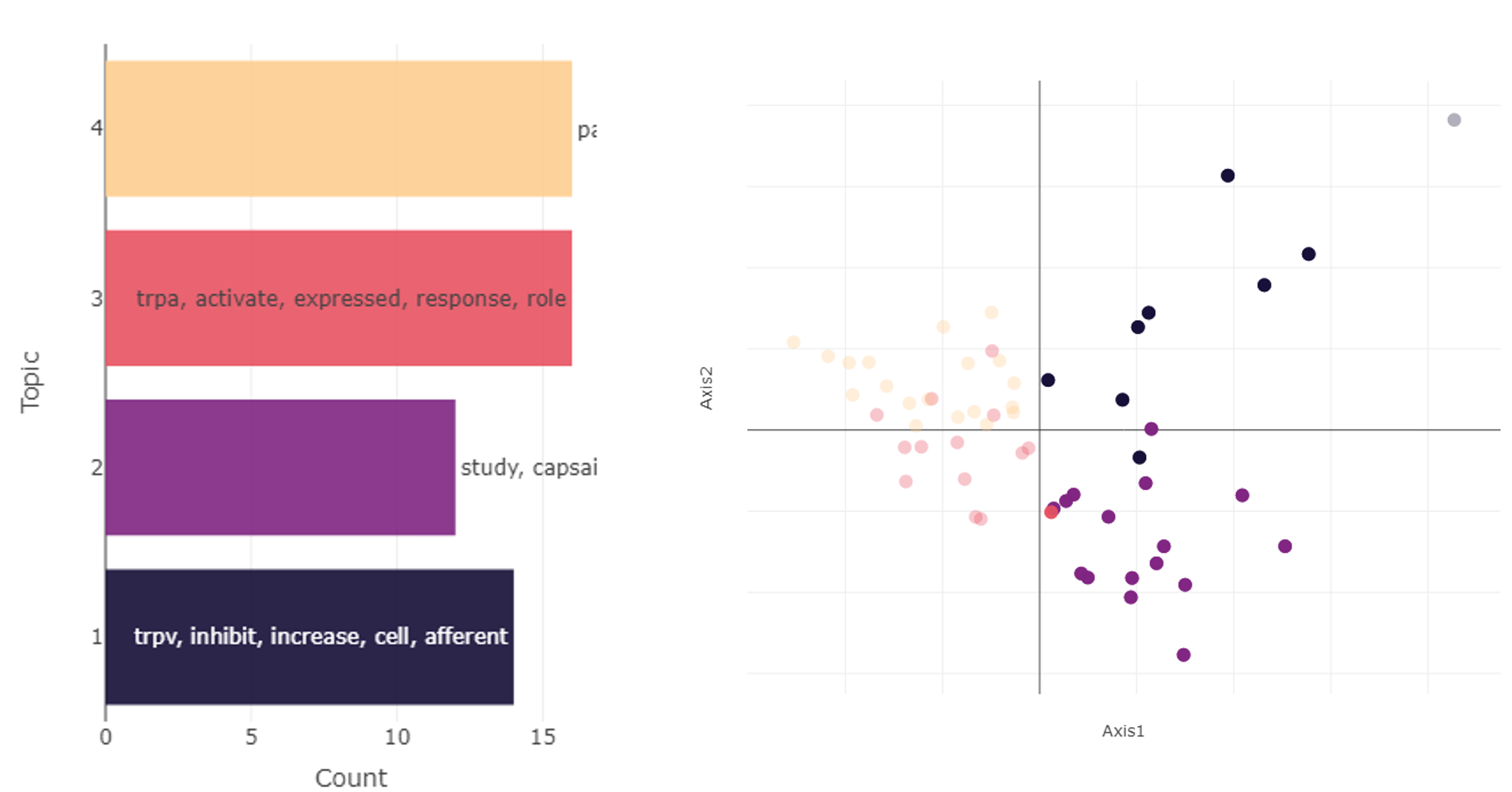

Supplement: Supplementary file 1 [file genes-14-00411-s001.zip › Figure S1.png]

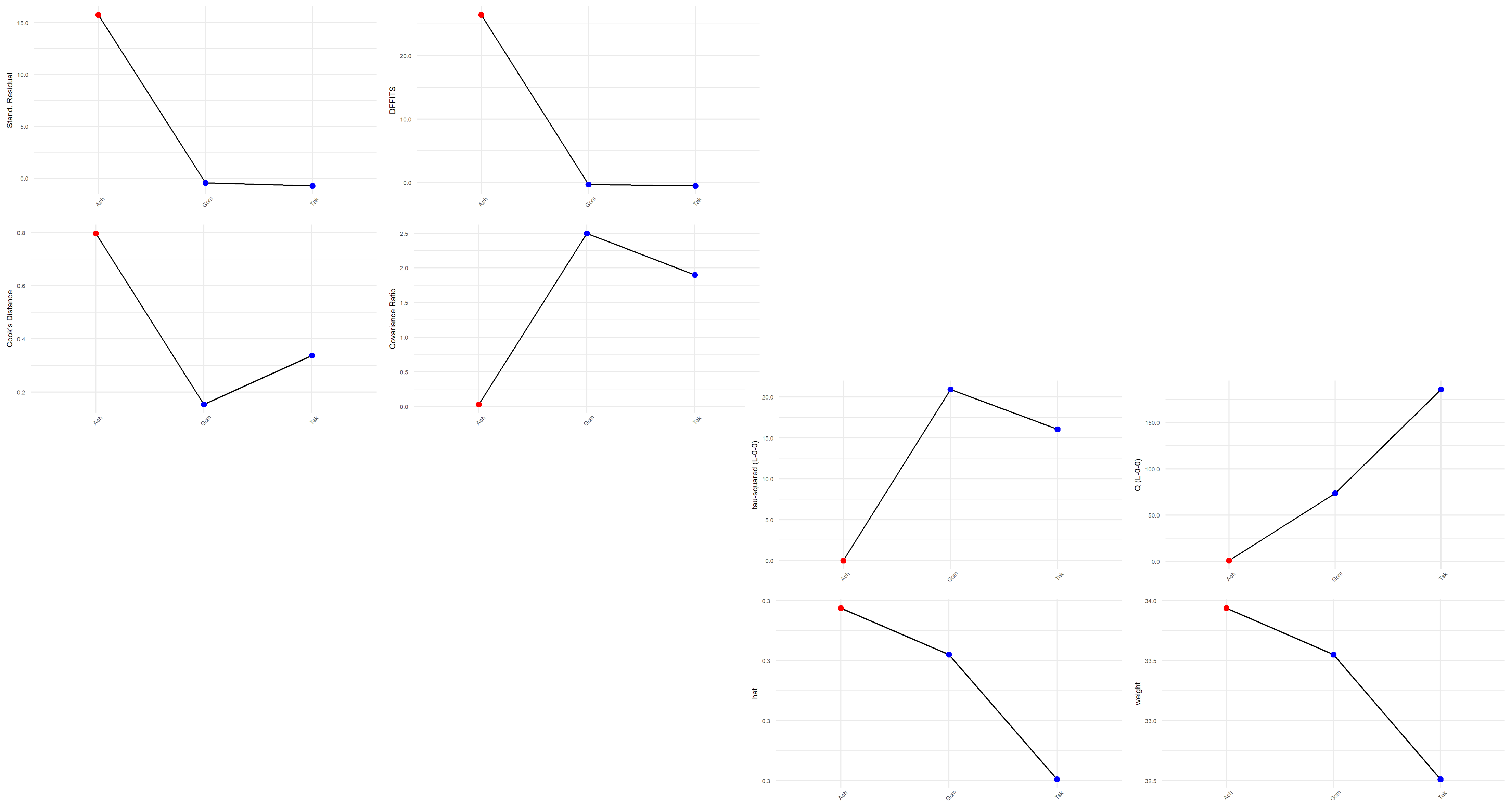

Supplement: Supplementary file 1 [file genes-14-00411-s001.zip › Figure S2.png]

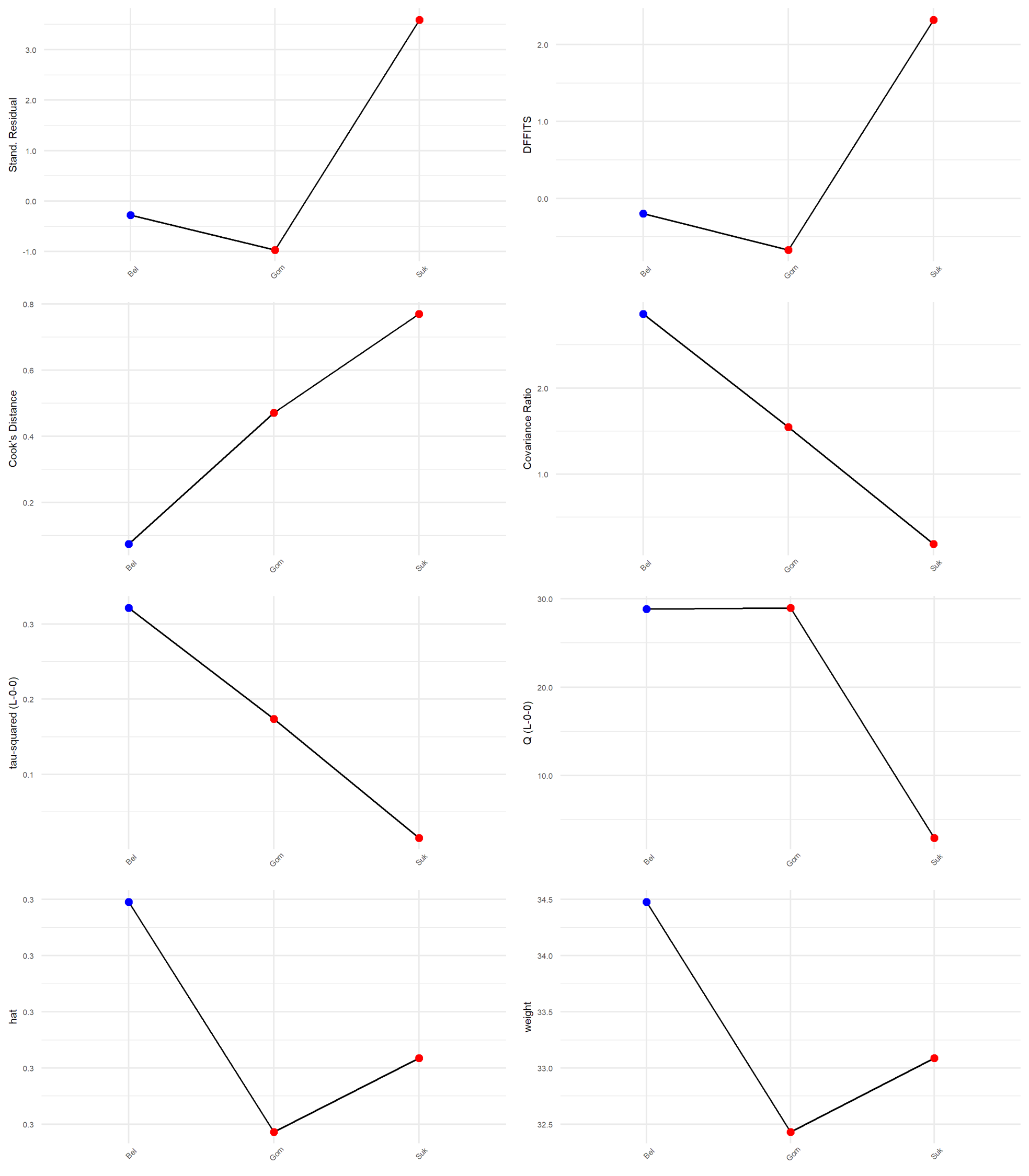

Supplement: Supplementary file 1 [file genes-14-00411-s001.zip › Figure S3.png]
